# Targeted chitosan nanobubbles as a strategy to down-regulate microRNA-17 into B-cell lymphoma models

**DOI:** 10.3389/fimmu.2023.1200310

**Published:** 2023-06-08

**Authors:** Sara Capolla, Monica Argenziano, Sara Bozzer, Tiziana D’Agaro, Tamara Bittolo, Luigina De Leo, Tarcisio Not, Davide Busato, Michele Dal Bo, Giuseppe Toffoli, Roberta Cavalli, Valter Gattei, Riccardo Bomben, Paolo Macor

**Affiliations:** ^1^ Department of Life Sciences, University of Trieste, Trieste, Italy; ^2^ Department of Scienza e Tecnologia del Farmaco, University of Turin, Turin, Italy; ^3^ Clinical and Experimental Onco-Hematology Unit, Centro di Riferimento Oncologico di Aviano (CRO)-Istituto di Ricovero e Cura a Carattere Scientifico (IRCCS), Aviano, Italy; ^4^ Department of Pediatrics, Institute for Maternal and Child Health, Istituto di Ricovero e Cura a Carattere Scientifico (IRCCS) Burlo Garofolo, Trieste, Italy; ^5^ Experimental and Clinical Pharmacology Unit, Centro di Riferimento Oncologico di Aviano (CRO)-Istituto di Ricovero e Cura a Carattere Scientifico (IRCCS), Aviano, Italy

**Keywords:** lymphoma, miR (microRNA), nanobubble, targeting antibody, animal model

## Abstract

**Introduction:**

MicroRNAs represent interesting targets for new therapies because their altered expression influences tumor development and progression. miR-17 is a prototype of onco-miRNA, known to be overexpressed in B-cell non-Hodgkin lymphoma (B-NHL) with peculiar clinic-biological features. AntagomiR molecules have been largely studied to repress the regulatory functions of up-regulated onco-miRNAs, but their clinical use is mainly limited by their rapid degradation, kidney elimination and poor cellular uptake when injected as naked oligonucleotides.

**Methods:**

To overcome these problems, we exploited CD20 targeted chitosan nanobubbles (NBs) for a preferential and safe delivery of antagomiR17 to B-NHL cells.

**Results:**

Positively charged 400 nm-sized nanobubbles (NBs) represent a stable and effective nanoplatform for antagomiR encapsulation and specific release into B-NHL cells. NBs rapidly accumulated in tumor microenvironment, but only those conjugated with a targeting system (antiCD20 antibodies) were internalized into B-NHL cells, releasing antagomiR17 in the cytoplasm, both *in vitro* and *in vivo*. The result is the down-regulation of miR-17 level and the reduction in tumor burden in a human-mouse B-NHL model, without any documented side effects.

**Discussion:**

Anti-CD20 targeted NBs investigated in this study showed physico-chemical and stability properties suitable for antagomiR17 delivery *in vivo* and represent a useful nanoplatform to address B-cell malignancies or other cancers through the modification of their surface with specific targeting antibodies.

## Introduction

microRNAs (miRNAs) were demonstrated to play important roles in many biological processes including apoptosis, proliferation, and differentiation of cells, being closely related to the pathogenesis, progression, and metastasis of different types of cancer (1–3). miRNAs themselves could act as tumor suppressors or oncogenes (onco-miRNAs), raising the possibilities to use them as therapeutic targets (4, 5). The expression of miRNAs can be modulated by two different approaches: miRNA mimics, which restore the lowered expression of tumor suppressors, and antagomiR molecules, which repress the regulatory functions of upregulated onco-miRNAs (6, 7).

A multitude of strategies have been developed to increase the efficacy of miRNAs *in vivo*, in order to avoid their rapid degradation in serum, elimination through kidneys and poor cellular uptake when injected as naked oligonucleotides (5). Delivery vectors must be highly efficient, safe, and easy to use. Until now, viral and non-viral carriers were produced. Methods based on viruses ensure high gene transfection efficiency, but their clinical applications are limited by the potential risk to induce neoplastic transformation of transfected cells and the lack of specificity for target tissues (8, 9). Non-viral vectors have recently received attention because they showed greater potential as gene delivery platforms; they are also not immunogenic, can be easily modified by targeting ligands and manufactured on a large-scale (9). Numerous non-viral vectors were developed and, in particular, those composed of positively-charged polymers [e.g. chitosan, polyethyleneimine, poly(amidoamine)], which electrostatically interact with DNA, siRNA and miRNA (5), represent a promising tool for transfection of targeted cells (10, 11).

B cell non-Hodgkin’s lymphoma (NHL), the most common hematological malignancy worldwide, refers to a diverse subtypes of B cell malignancies, including either indolent cases, e.g. follicular lymphoma and small lymphocytic lymphoma/chronic lymphocytic leukemia (SLL/CLL), or aggressive subtypes, such as diffuse large B-cell lymphoma (DLCBL) and Burkitt lymphoma (BL) (12, 13). The standard first-line therapy for aggressive lymphomas, based upon polychemotherapy plus the anti-CD20 antibody rituximab, yields about 60% of responders (14, 15); however, for patients who do not respond to first-line therapies and failed second options, representing about the 30-40% of cases, treatment outcomes are very poor (16, 17).

The management of relapsed/refractory elderly and immunocompromised patients represents a great challenge, and approaches addressing new therapeutic targets represent an important medical need. In the present study, we investigated the ability of antiCD20-targeted chitosan nanovesicles, called nanobubbles (NBs), to deliver *in vitro* and *in vivo* anti-miRNAs as a therapeutic option for BL treatment. Anti-CD20 antibodies were already demonstrated to specifically mediate the binding of nanoparticles (NPs) to target cells (18–22), supporting the forcefulness of this targeting mechanism. For what concerns the payload, it represents a novelty in the field; previously published NPs against BL were based just on the encapsulation of chemotherapeutics such as vincristine (23), daunorubicin or doxorubicin (21, 24). In our system, we took advantage of RNA interference (RNAi) strategy for improving the therapeutic treatments.

Members of miR-17~92 cluster were demonstrated to be upregulated by several subtypes of B-NHL cells, included BL, often characterizing subgroups with worse prognosis and with peculiar clinico-biological features (25, 26). In fact, the over-expression of miR-17 was shown to correlate to a poorer 5-year overall survival of BL-affected patients (27), representing a good therapeutic target. Thus, a RNA molecule, antagomiR17, was synthesized and characterized for the capacity to induce the degradation of miR-17 and to affect the growth of miR-17-overexpressing tumor B cells (28). AntagomiR17-loaded antiCD20-conjugated NBs were demonstrated to selectively address miR-17-expressing lymphoma cells both *in vitro* and *in vivo*, showing an effective and safe therapeutic effect.

## Materials and methods

### Materials

All materials used were of analytical grade and purchased from Sigma-Aldrich (St. Louis, MO, USA), unless otherwise stated. Soybean lecithin (Epikuron 200^®^) was kindly supplied by Cargill (Hamburg, Germany). Chitosan low molecular weight (degree of deacetylation 75–85%, 50–190 KDa) was used.

### Preparation of NB formulations

Chitosan shelled/perfluoropentane-cored NB formulations were prepared according a tuned manufacturing method elsewhere described (10). Briefly, a nanoemulsion was obtained adding an ethanol solution containing Epikuron^®^200 and palmitic acid (1% w/v) and perfluoropentane to ultrapure water. Then, this system was homogenized using an Ultra-Turrax SG215 homogenizer (IKA, Konigswinter, Germany). Subsequently, a chitosan solution (2.7% w/v, pH 4.5) was drop-wise added under mild magnetic stirring to obtain the chitosan-shelled blank NBs.

Double-labelled fluorescent NB formulations were produced following the above method, but encapsulating coumarin-6 in the NB perfluoropentane core (50 μg/ml, Sigma Aldrich, St. Louis, MO, USA) and binding cyanine 5.5 (Cy5.5, 10 nmol/ml, FluoroLink™ Cy5.5 Monofunctional Dye, GE Healthcare Bio-Sciences AB, Uppsala, Sweden) to the NB chitosan shell. The labeling of NBs with Cy5.5 was obtained by incubating the pre-formed chitosan-shelled NBs with a Cy5.5 DMSO solution under magnetic stirring at room temperature in the dark for 1 h. The amount of bound Cy5.5 was quantified by spectrophotometric methods using the molar extinction coefficient of 250,000 M^-1^ cm^-1^ at 678 nm.

Targeted NB formulations were prepared by the chemical conjugation of anti-CD20 antibodies (Rituximab, Roche, Milan, Italy, 100 µg/ml) to the chitosan shell of NBs. The anti-CD20 antibodies were oxidized by periodate oxidation and conjugated by reductive amination to chitosan amino groups (29). Targeted and untargeted NB formulations loaded with antagomiR17 were developed by incorporating the antagomiR17 (5’-mC mU mA mC mC mU mG mC mA mC mU mG mU mA mA mG mC mA mC mU mU mU mG-3’, Integrated DNA Technologies) within the chitosan shell. To obtain the antagomiR17 encapsulation, an aqueous solution of antagomiR17 was drop-wise added to the NBs to obtain the concentration of 0.8 mg/ml and incubated under magnetic stirring at 4°C for 30 minutes. Finally, a Pluronic F68 solution (0.01% v/v) was added to NBs as a stabilizing layer.

The composition of different NB formulations prepared were reported in **Table 1**.

**Table 1:** Composition of the NB formulations.

Sample	Cy5.5 dye	6-coumarin	anti-CD20 antibody	antagomiR17
Blank NB	–	–	–	–
NB0	10 nmol/ml	50 μg/ml	–	–
NB1	10 nmol/ml	50 μg/ml	100 μg/ml	–
NB2	–	–	100 μg/ml	800 μg/ml
NB3	–	–	–	800 μg/ml

### Physicochemical characterization of NB formulations

The average diameter, polydispersity index (PDI) and zeta potential of NBs were determined by dynamic light scattering (DLS) using a 90 Plus instrument (Brookhaven, NY, USA). The analyses were performed at a scattering angle of 90°C and a temperature of 25°C, using a NB suspension diluted with deionized distilled water. For zeta potential determination, samples of diluted NB formulations were placed in the electrophoretic cell where an electric field of approximately 15 V/cm was applied. Bound antibody concentration was measured by the Pierce™ BCA protein assay kit (Thermo Scientific, Rockford, IL USA). One mL of the BCA reagent was added to 100 µl of the sample and reacted for 30 min at 37°C. After cooling to room temperature, the chromogenic product was measured by an UV-Vis spectrophotometer at 562 nm. The anti-CD20 antibody concentration was calculated referring to a calibration curve obtained analyzing antibody standard solutions in the concentration range between 0 – 200 μg/ml. The antagomiR17 concentration in the NBs was determined by spectrophotometric analysis using an UV–visible spectrophotometer (VICTOR X; Multilplate Reader, Perkin Elmer, Waltham, MA, USA) set at the wavelength of 260 nm. The complete encapsulation of antagomir17 in NBs was investigated from the spectrophotometric quantification of free antagomir17 after NB centrifugation (15,000 rpm, 15 min, 4°C) using an Amicon®Ultra-0.5 centrifugal filter unit (Sigma Aldrich, St. Louis, MO, USA).

Nanobubble morphology was observed by Transmission Electron Microscopy (TEM) using a Philips CM10 (Eindhoven, NL) instrument. The diluted nanobubble in aqueous suspensions were sprayed on Formwar-coated copper grid and air dried before observation.

### *In vitro* release studies

To investigate the *in vitro* release of antagomiR17 from NBs in cellular environment, NB2 and NB3 formulations were incubated in simulated cytosol (142 mM KCl, 5 mM NaCl, 5 mM MgCl_2_, 25 mM Hepes-KOH (pH 7.2), 1 mg/ml BSA) at a 1:4 v/v ratio over time (6, 24, 48 h). At the fixed times, gel retardation assay was performed. The samples were loaded into agarose gel (3% w/v), stained with an ethidium bromide solution (0.5 μg/ml). The electrophoresis ran in TAE buffer 1X (40 mM Tris base, 20 mM acetic acid and 1 mM EDTA; pH 8.0) at 120 V for 25 min. Free antagomiR17 (200 μg/ml) was used as a positive control. The banding pattern was visualized using an ultraviolet transilluminator and photographed with a Polaroid camera.

### Cells and antibodies

The B-NHL cell line BJAB representing BL (high miR17 expression) and the CLL/SLL cell line MEC-1 (low miR17 expression) were cultured in RPMI-1640 medium (Sigma-Aldrich, Milan, Italy) supplemented with 10% fetal bovine serum (FBS; Gibco, Invitrogen, Milan, Italy), L-glutamine and a mixture of penicillin and streptomycin (Sigma-Aldrich, Milan, Italy).

Rituximab is an anti-CD20 antibody used as a targeting agent bound on NBs and derived from the clinic.

For immunofluorescence, rat anti-mouse CD68 antibody and the secondary rabbit anti-rat IgG antibody conjugated with TRITC were purchased from Biorad (Milan, Italy) and Sigma-Aldrich (Milan, Italy), respectively.

### Binding and internalization of NBs into B-cells

The binding/internalization of NBs was assayed incubating BJAB cells (5x10^5^) with 4µl of NB0 or NB1 for increasing amount of time (15 min to 4 h). After incubation, cells (10,000 events) were acquired by the flow cytometer BD FACSCanto™ II (Beckton-Dickinson, Franklin Lakes, NJ, USA) and analyzed with the FACSDiva software (Beckton-Dickinson, Franklin Lakes, NJ, USA). For internalization studies, 50 µl of the flow cytometry sample were cytocentrifuged with the Shandon CytospinIII Cytocentrifuge (GMI, Ramsey, MN, USA) at 200 rcf for 5 min on positive-charged glasses (Bio-Optica, Milan, Italy). Membranes and nuclei of cells were labeled with Vybrant™ DiI cell-labeling solution (ThermoFisher Scientific, Waltham, MA, USA) and DAPI (Sigma-Aldrich, Milan, Italy), respectively. Samples were analyzed with the confocal microscope Eclipse te300 (Nikon Corporation, Tokyo, Japan). Images analysis and fluorescence quantification were performed using the Fiji software (Fiji-NIH).

### Internalization studies of NBs in human macrophages

THP-1 were cultured in RPMI-1640 medium (Sigma-Aldrich, Milan, Italy) supplemented with 10% fetal bovine serum (FBS; Gibco, Invitrogen, Milan, Italy), L-glutamine and a mixture of penicillin and streptomycin (Sigma-Aldrich, Milan, Italy). The *in vitro* differentiation of THP-1 to macrophages was made incubating cells with 100ng/ml of PMA (Phorbol 12-Myristate 13-Acetate, Sigma-Aldrich, Milan, Italy) for 72 h. Cells (1x106) were incubated with NB0 or NB1 for increasing amount of time (10, 20, 30 min or 2h) in RPMI at 37°C or 4°C. After the incubation with NBs, cells were washed three times with PBS to remove unbound particles and detached by trypsin-EDTA (Sigma-Aldrich, Milan, Italy) for 5 min at 37°C. Cells (10,000 events) were acquired by FACSCalibur (Beckton-Dickinson, Franklin Lakes, NJ, USA) flow cytometer and data were analyzed by CELLQuest software (Beckton-Dickinson, Franklin Lakes, NJ, USA).

### Transfection and quantitative-real-time polymerase chain reactions

Four micrograms of antagomiR17 (Integrated DNA Technologies) were added to 7.5 x 10^5^ BJAB cells resuspended in 1 ml of culture medium. Total RNA was extracted from cells and from frozen animal tissues (50 µm-slices) using the TRIZOL Reagent (Thermo Fischer, Waltham, MA, USA).

The expression of miR-17 and the control *RNU6B* was assessed using a standard TaqMan MicroRNA assay kit (Thermo Fischer) according to the manufacturer’s instructions and as previously described (30). Briefly, microRNA was reverse transcribed to cDNA using gene-specific primers and the relative amount of each microRNAs was computed using the equation 2^-ΔCt^, where ΔCt=(Ct _microRNA_ – Ct _RNUB6_). Fold-change between classes was calculated as reported (30). All qRT-PCR experiments were performed on an CFX96 System (BioRad, Hercules, CA, USA).

### Animals

Female severe combined immunodeficiency (SCID) mice (4-6 weeks of age) were purchased from Envigo (Udine, Italy) and maintained under pathogen-free conditions. All the experimental procedures involving animals were done in compliance with the guidelines of the European (86/609/EEC) and the Italian (D.L.116/92) laws and were approved by both the Italian Ministry of Health (Prot. N° 507/2016-PR) and the Administration of the University Animal House.

The development of a BL tumor was induced as previously described (18); briefly, 2x10^6^ BJAB cells in saline (0,1ml) were injected intraperitoneally in SCID mice and a tumor mass developed in the site of injection in 20-25 days. Similarly, 10^7^ MEC1 cells in saline (0,1ml) were injected intraperitoneally in SCID mice to induce a CLL/SLL tumor mass (28).

### *In vivo* biodistribution analysis

When the tumor mass reached an average volume of 250-300 mm^3^, 0.6 nmol of Cy5.5 free or conjugated to NB0 and NB1 were injected in tail vein of mice. Animals were shaved before acquisition to reduce the scattering of the signal from hair, as previously described (*in vivo* biodistribution and lifetime analysis of cy5.5-conjugated rituximab in mice bearing lymphoid tumor xenograft using time-domain near-infrared optical imaging) (31). *In vivo* biodistribution studies were performed before NBs injection (background) and after 1, 24, 48, 72, and 96 h by IVIS Lumina III (PerkinElmer, Milan, Italy). At the end of the analysis, mice were euthanized, and explanted organs were analyzed by IVIS Lumina III. Liver and tumor masses were washed in PBS, embedded in OCT (Optimal Cutting Temperature) compound embedding medium (Miles Inc., Diagnostics Division, Elkhart, IN, USA) and snap-frozen at -80°C.

### Confocal microscopy of tissues

Tumor and liver sections of 30µm were cut from frozen organs with a cryostat at -20°C, and treated as previously described (32). Slices were firstly fixed with PFA 4% for 20 min and rehydrated for 5 min with PBS at room temperature. Liver slices were incubated with 2% of rabbit serum (Dako, Santa Clara, CA, USA) in PBS for 30 min and then incubated with rat anti-mouse CD68 antibody for 1 h at room temperature. After 3 washes with PBS (30 min), liver slices were incubated with the secondary TRITC-conjugated rabbit anti-rat IgG antibody for 1 h and additionally washed three times with PBS for 10 min. Liver and tumor slices were finally incubated with DAPI (Sigma-Aldrich, Milan, Italy) for 5 min at room temperature. The excess of DAPI was eliminated by additional washes with PBS (15 min).

Images were acquired with the confocal microscope Eclipse te300 (Nikon Corporation, Tokyo, Japan) and analyzed using Fiji software.

### Therapeutic efficacy of antagomiR17 and NBs

When CLL/SLL tumor mass reaches the volume of 300 mm^3^, mice were intratumorally injected with physiologic solution (untreated mice), antagomiR17 free (120 µg) or NB2 (corresponding to 120 µg of antagomiR17) for 4 times (day 0, 3, 10, 16). Before injection, free antagomiR17 was diluted in a solution of saline and Lipofectamine2000 (Invitrogen, Milan, Italy) at a 1:1 v/v ratio and incubated for 10 min at room temperature. AntagomiR17 requires Lipofectamine to maximize the entrance in cancer cells, even if they are locally injected; this approach was previously used (28) and here compared with the capacity of polymeric nanostructures to transfer antagomiR17 inside cancer cells.

BL-bearing mice received intraperitoneal injections of antagomiR17 (120 µg) loaded inside NB2 or NB3, 3 times a week for 8 times.

Tumor size was assessed every two or three days by caliper measurement. Tumor volume was calculated as follow: volume = D x d^2^ x π/6, where D and d are the longer and the shorter diameters of the mass, respectively.

### Statistical methods

All data has been checked for normal distribution and analyzed in GraphPad Prism.8.0.0. Results from at least three independent experiments were reported as means ± standard deviations or mean ± standard error mean. Data were analyzed by the two-tailed Student’s t test. Statistical analysis of tumor mass was performed by two-way ANOVA test. *P*-values <0.05 or less were considered statistically significant. G*Power statistical power analysis program has been initially used to set *in vivo* experiment and a statistical power of 80% using data already collected by our group with these animal models.

## Results

### NBs characterization

Chitosan-shelled/perfluoropentane-cored NBs were initially developed. Different types of NBs were produced starting from blank NB formulations, comprising only a chitosan shell on the surface and perfluoropentane in the core. In details, fluorescent NB0 were prepared loading coumarin-6 in the perfluoropentane core and binding Cy5.5 to the chitosan shell; NB1 derived from the conjugation of anti-CD20 antibodies on NB0 shell. Moreover, targeted or untargeted antagomiR17-loaded NB formulations, NB2 and NB3 respectively, were produced loading antagomiR17 within the chitosan shell (**Table 1**).

**Figure 1:**
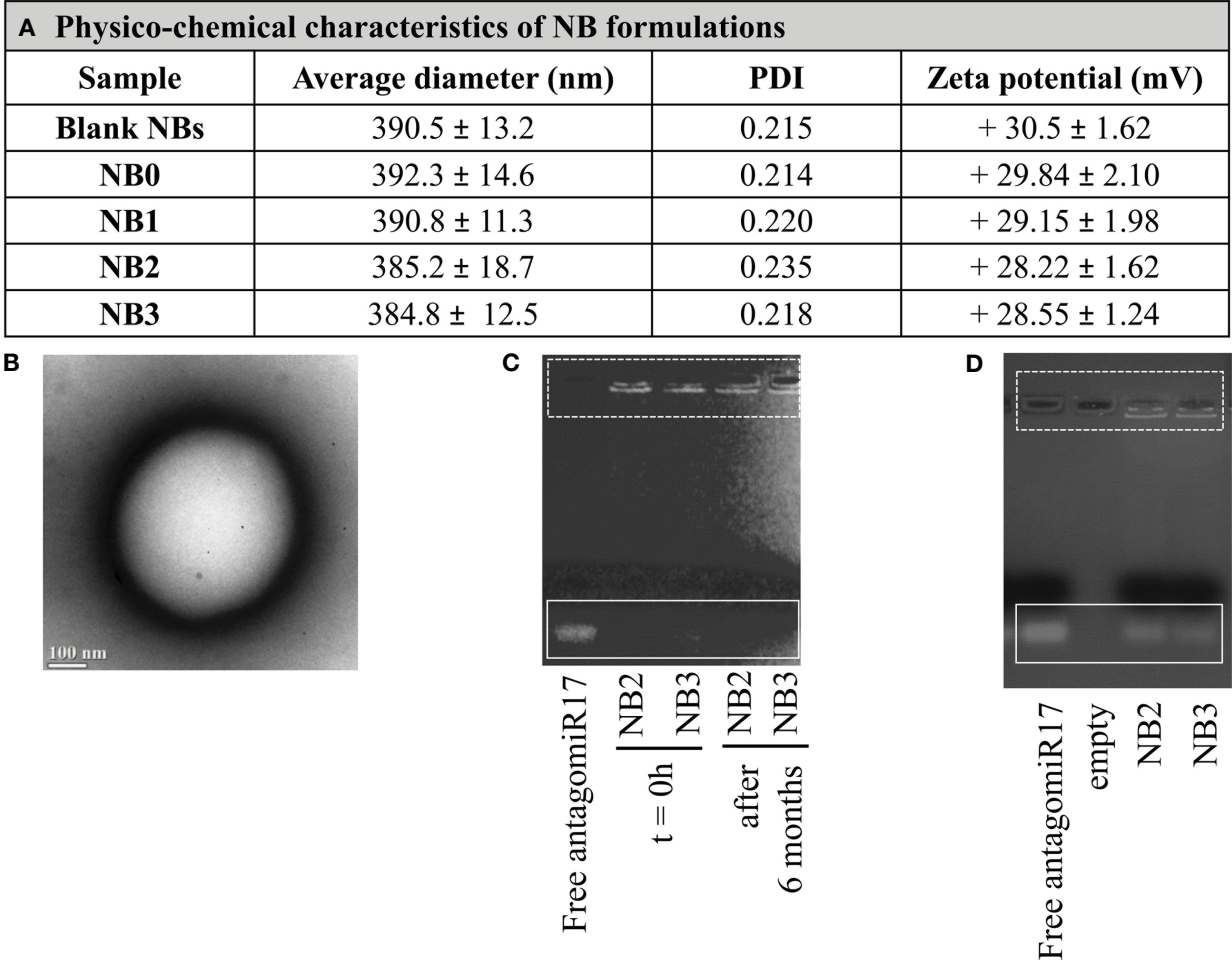
Nanobubbles characterization. **(A)** Physicochemical characteristics of NB formulations. **(B)** TEM image of NBs (scale bar 100 nm). **(C)** AntagomiR17 incorporation in NBs and NB stability over time. **(D)** *In vitro* release of antagomiR17 from targeted and untargeted antagomiR17-loaded NBs (NB2 and NB3, respectively).

All the NB formulations showed average sizes lower than 400 nm and a positive surface charge (around +30 mV). The average diameter, polydispersity index (PDI), zeta potential values of the different types of NBs are reported in **Figure 1A**. The sizes and surface charge of NBs showed only negligible modifications after antagomir17 loading. These data underline that the incorporation of antagomiR17 in the NBs did not alter their structure or change their physicochemical parameters. A representative image of the morphology of chitosan NBs is shown in **Figure 1B**: a spherical shape and a well-defined core-shell structure were observed. The capacity of NBs to load and retain the antagomiR17 was also confirmed by the gel retardation assay, where the antagomiR17 band was not detected when it is incorporated in NB2 and NB3 formulations (**Figure 1C**).

NB formulations showed a good stability over time, when stored at 4°C for 6 months. Indeed, no size increase, zeta potential modification or NB aggregation occurred (data not shown). In addition, the *in vitro* stability of antagomiR17-loaded NBs was evaluated by gel retardation assay after 6 months. Both the formulations NB2 and NB3 fully retained the antagomiR17, as shown in **Figure 1C**, confirming the physical stability of the nanosystem up to 6 months.

The *in vitro* release of antagomiR17 from the NB2 and NB3 was evaluated in simulated cytosol over time to mimic the intracellular environment. Naked antagomiR17 (2 µg) migrated through the gel towards the positive pole and a band was visible on the gel. On the contrary, after 6 h of incubation, a band was observed for both the formulations, indicating that antagomiR17 can be released in cell cytoplasm after internalization of nanoparticles (**Figure 1D**). To note, some fluorescence is still visible in the wells suggesting an incomplete *in vitro* release of antagomiR17 after 6 h.

### Anti-CD20 antagomiR17-loaded NBs affect miR-17 levels *in vitro*

**Figure 2:**
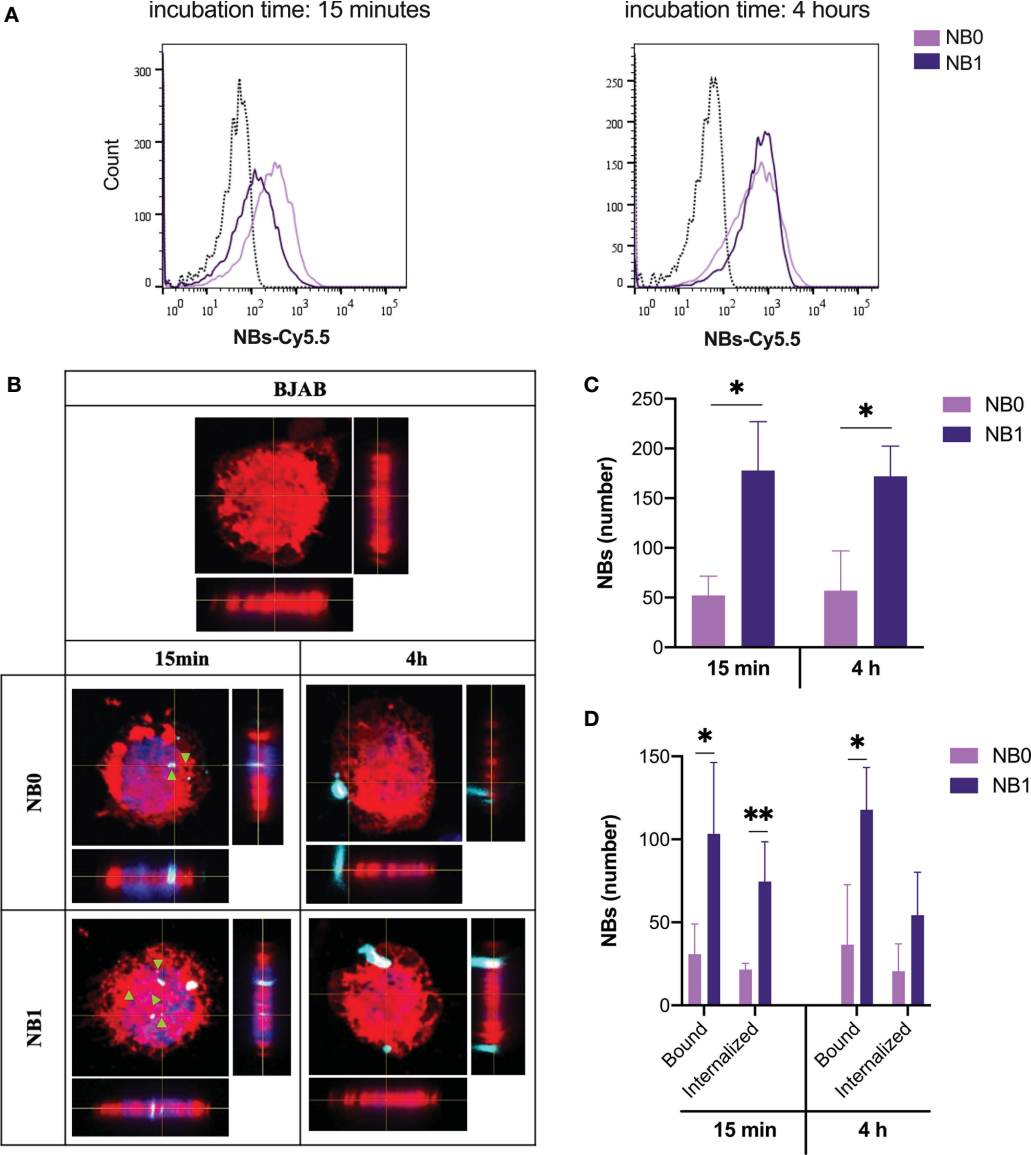
NBs bind and internalize in tumor B cells. **(A)** Cytofluorimetric analysis of BJAB cells incubated with Cy5.5-conjugated NBs at 37°C. **(B)** Analysis of NBs internalization by confocal microscopy labeling cell membrane with Vybrant™ DiI (red), nuclei with DAPI (blue) and NBs with Cy5.5 (light blue). Original magnification 630X. Green arrows highlight internalized particles. **(C)** Quantification of the total amount of NBs detected on cells **(D)** Quantification of bound and internalized NBs in BJAB cells. Data are showed as mean ± SD. * p-value <0.05; ** p-value <0.005. NB0: untargeted NBs; NB1: antiCD20-conjugated NBs.

The contribution of the anti-CD20 antibodies, conjugated on the surface of NBs, to the binding and the internalization of nanosystems, was evaluated incubating NB0 and NB1 with CD20-expressing cancer B cells (BJAB). At short incubation time (15 min), NB0 interact with a higher number of cells with respect to NB1 (NB0 vs NB1: 66.8% vs 36.3%) but this difference was completely flattened at longer incubation times (4 h, NB0 vs NB1: 79.9% vs 66%, **Figure 2A**).

When visualized by confocal microscopy, two different binding patterns were evidenced. At short incubation time, both NBs appeared as small spots deposited around and inside cells while after 4 h just big agglomerates of NBs were visible (**Figure 2B**). Additionally, on the contrary to flow cytometric analysis, among positive cells, the total amount of detected NB1 was significantly higher than NB0, as suggested by NBs quantification through confocal microscopy (**Figure 2C**). The same pattern was detected if bound and internalized NBs are considered separately. In details, a significantly higher number of NB1 than NB0 was demonstrated to be bound after short and long incubation times; for what concerns internalized particles, a statistical significance between NB1 and NB0 was detected just after an incubation of 15 min (**Figure 2D**).

**Figure 3:**
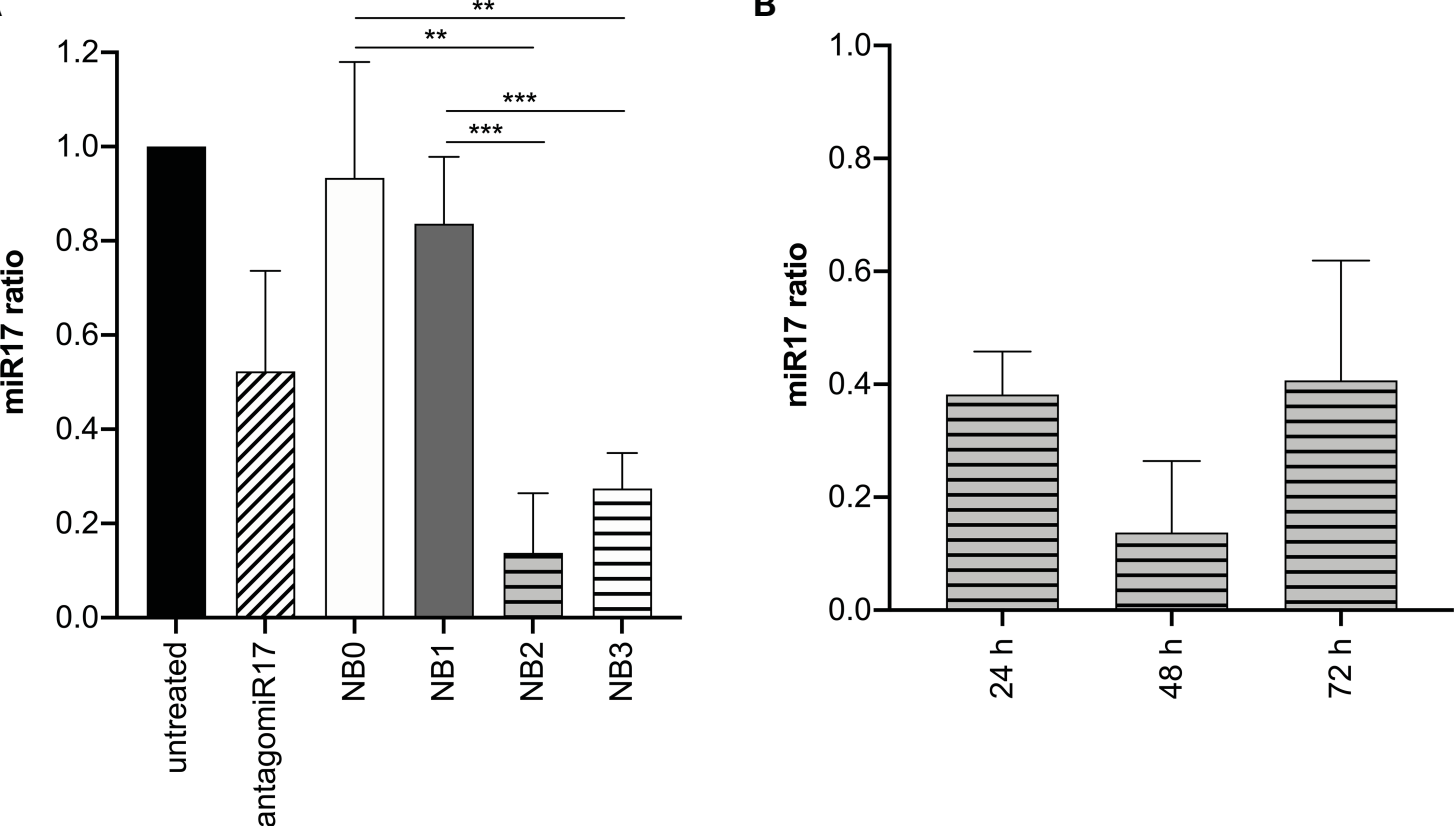
antagomiR17-loaded NBs reduces miR-17 levels in tumor B cells. **(A)** Analysis of miR-17 levels in BJAB cells treated with antagomiR17 free or loaded in NBs. **(B)** Analysis of miR-17 levels in BJAB cells treated with NB2 for 24, 48 or 72 h. NB0: empty untargeted NBs; NB1: empty targeted NBs; NB2: antagomiR17-loaded targeted NBs; NB3: antagomiR17-loaded untargeted NBs. ** p-value <0.005; *** p-value <0.0005.

To evaluate the capacity of NBs to reduce the amount of basal miR-17 levels after internalization, BJAB cells were treated with free antagomiR17 and with all the NBs formulations. As expected, empty NBs such as NB0 and NB1 did not affect miR-17 expression levels; on the contrary, antagomiR17-loaded NB2 and NB3 significantly decreased its cellular concentration (**Figure 3A**), suggesting that encapsulated antagomiR17 is still active and released in the tumor cells, but also that the level of miR-17 is modulated just by encapsulated antagomiR17 and not by a polymers/perfluoropentane/antiCD20-mediated effect. To note that the absence of a significant difference in the effect induced by NB2 and NB3 confirms what previously shown: for long incubation times *in vitro*, targeted and untargeted NBs are similarly internalized. Moreover, in a time-course analysis, NB2 showed the best interfering effect after 48 h of incubation with cells, while at 72 h miR-17 expression started to be restored (**Figure 3B**).

### Local injection of antagomiR17-loaded NBs arrests tumor growth by decreasing miR-17 expression *in vivo*

To evaluate a potential effect *in vivo*, repeated intratumor treatments were initially evaluated, as previously performed with free antagomiR17 in a localized model of B-cell malignancy (28).

**Figure 4:**
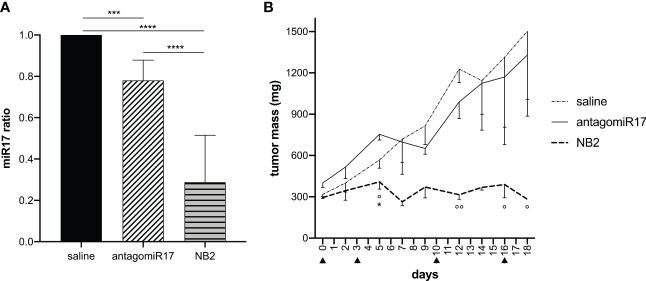
Local treatment with NB2 reduces miR-17 levels in tumor B cells and inhibits tumor growth. Tumor-bearing mice received intratumoral injection of free antagomiR17 or antagomiR17-loaded targeted NBs (NB2). **(A)** The expression of miR-17 was measured *ex vivo* by Real-Time PCR (n = 3) at the end of the study. Data are showed as mean ± SD. *** p-value <0.0005 **** p-value <0.0001. **(B)** Tumor mass dimension was measured during treatments (▲). Data are showed as mean ± SEM. Two-way ANOVA was used. AntagomiR17 vs saline: * p-value <0.05; NB2 vs saline: ° p-value <0.05, °° p-value <0.005.

Free antagomiR17, injected intratumorally with the same previously tested protocol (28), induced a significant reduction of miR-17 level *in vivo* (**Figure 4A**). However, free antagomiR17 did not affect tumor growth (**Figure 4B**). On the contrary, antagomiR17-loaded NB2 strongly down-regulated miR-17 expression in the tumor mass after local repeated injections (**Figure 4A**), also inducing a complete arrest of tumor growth *in vivo*, particularly evident after the second injection and maintained during the treatment (**Figure 4B**).

### NBs addressed B-cell tumor masses and were eliminated through liver

Macrophages, and in particular Kupffer cells, represent one of the most important clearance routes of NPs (33). In order to characterize the interaction of the nanosystems used in this study with macrophages, NB0 and NB1 were incubated *in vitro* with the human monocyte cell line model THP-1 cells after activation through PMA. This analysis confirmed the interaction of both NBs with activated monocytes without any statistical difference. The interaction of NBs with these cells (gating strategy is represented in Additional File 1: **Supplementary Figure 1A**) was significantly inhibited by the decrease of temperature during incubation, as shown in **Supplementary Figure 1**, analyzing both percentage (Additional File 1: **Supplementary Figure 1B**) or fluorescence intensity (Additional File 1: **Supplementary Figure 1C**) of interacting cells; this suggests the implication of membrane movements typical of active phagocytosis.

**Figure 5:**
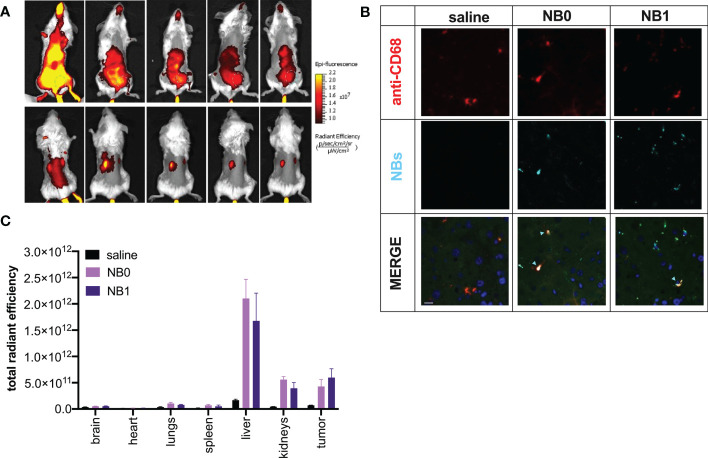
NBs mainly distributed in tumor mass, liver and kidneys. Cy5.5-labeled NBs were injected i.v. in tumor-bearing mice. **(A)** *In vivo* distribution studies were performed for 96 h showing NBs accumulation mainly in liver, tumor mass (from supine position – upper panel. Tumor: green dashed line-circle) and kidneys (from prone position – upper panel). **(B)** NBs elimination by the liver was confirmed by immunofluorescence analysis. Macrophages were visualized using anti-mouse CD68 and TRITC-conjugated secondary antibody (red fluorescence), cell nuclei by DAPI (blue fluorescence) and NBs by their near-infrared fluorescence (light blue); green fluorescence represents tissue autofluorescence. Samples were analyzed by confocal microscopy. Scale bar: 10µm. Light blue arrows highlight NBs/macrophage co-localization. **(C)** Data were confirmed *ex vivo* analyzing NBs fluorescence in different organs at the end of the experiment. Data are shown as mean ± SEM. n = 3. NT: untreated mice; NB0: untargeted NBs; NB1: antiCD20-conjugated NBs.

The contribution of the liver in NBs elimination was confirmed also through *in vivo* biodistribution analysis (**Figure 5A** - upper panels); in fact, NBs were mostly eliminated by this organ and Kupffer cells (CD68+) were in part responsible for this pattern, as demonstrated by *ex vivo* immunofluorescence analysis of liver sections (**Figure 5B**). In addition, biodistribution analysis also suggested an unexpected presence of NBs in the kidneys; fluorescence was revealed in these organs starting from 1 h post injection to the end of the observation (**Figure 5A** – lower panels).

*Ex vivo* analysis, shown in **Figure 5C**, confirmed these data: liver is the organ causing the main accumulation/elimination of the nanosystems and about 60% of residual fluorescence was documented in this organ while about 20% was revealed in the kidneys. The signal reported in the latter was demonstrated to be not related to the presence of free Cy5.5, contaminant of the sample, or released from the nanosystems; indeed, the injection of free Cy5.5 in mice clearly showed a different distribution pattern, with a prevalent elimination 24 h post-injection and did not localize into kidneys but accumulate in the bladder (Additional file 1: **Supplementary Figure 2**).

**Figure 6:**
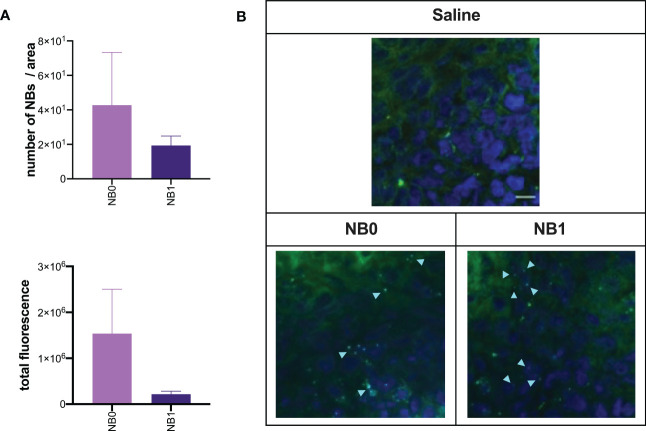
Targeted and untargeted NBs localized in the tumor mass. At the end of *in vivo* biodistribution studies, tumor sections were analyzed by confocal microscopy. **(A)** The amount of fluorescence and the number of NBs was quantified by Fiji software and expressed as mean ± SEM. **(B)** Cy5.5-labeled NBs (light blue) were also visualized by confocal microscopy; nuclei were stained with DAPI (blue fluorescence), green fluorescence represents autofluorescence of tumor tissue. Scale bar: 10µm. NB0: untargeted NBs; NB1: antiCD20-conjugated NBs.

*In vivo* biodistribution studies clearly demonstrated the presence of NBs in the tumor mass. Their localization in this microenvironment was not mediated by a local presence of macrophages because these cells were absent in this tissue (data not shown). The presence of both NB0 and NB1 in the tumor mass was detected without a significant difference between particles (**Figure 5A**). This was also confirmed by the *ex vivo* quantification of the fluorescence and the number of NBs in these samples, after confocal microscopy images acquisition (**Figure 6A**). Despite the absence of differences, microscopy images showed a different pattern of NBs in the tumor tissue: NB0 appeared as bigger clusters while NB1 were more scattered and smaller (**Figure 6B**).

### AntagomiR17-loaded targeted NBs arrest tumor growth *in vivo*

**Figure 7:**
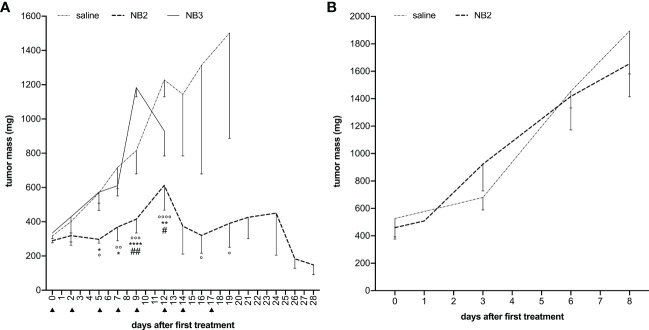
Anti-CD20 NB2 treatment reduces tumor mass neutralizing miR-17 activity. **(A)** Human-mouse model developed injecting BJAB cells in SCID mice received i.p. injections (▲) of antagomiR17-loaded targeted (NB2) or untargeted NBs (NB3). Tumor mass was measured, and data are showed as mean ± SEM. NB2 vs NB3: * P-value <0.05; ** P-value <0.005, **** P-value <0.0001; NB2 vs NT: ° P-value <0.05, °° P-value <0.005, °°° P-value <0.0005, °°°° P-value <0.0001. NB2 vs NB3: # P-value <0.05, ## P-value <0.005. **(B)** Human-mouse model developed injecting MEC-1 cells (expressing low levels of miR-17) in SCID mice received i.p. injections of antagomiR17-loaded targeted (NB2) or saline. Tumor mass was measured, and data are showed as mean ± SEM.

In order to characterize the *in vivo* effect of antagomiR17, tumor-bearing animals were treated via intraperitoneal injections of NB2 or NB3 for 18 days; animals received the nanosystems 3 times per week, exploiting the fast accumulation of the NBs (**Figure 5**) and their pick of efficacy after 48 h (**Figure 3**). The presence of the anti-CD20 antibody on the surface of nanosystems was demonstrated to be fundamental for the efficacy of antagomiR17-loaded nanoparticles. As a matter of fact, NB2 completely arrested tumor growth and all animals survived at the end of the study. On the contrary, NB3 (antagomiR17-loaded nanoparticles without a targeting agent) were ineffective, showing similar tumor growth with respect to saline-treated mice; all the animals treated with saline or NB3 have been sacrificed in less than 20 days after the first treatment because of the dimension of the tumor mass (**Figure 7A**).

To demonstrate that the therapeutic effect of the targeted nanosystems was dependent only by the presence of antagomiR17 and not induced by the anti-CD20 antibody, polymers or perfluoropentane, the *in vivo* experiments were then performed in a second human/mouse model of B-cell malignancy; animal were challenged with a specific population of MEC1 cells, expressing low levels of miR-17. In this context, the effect of NB2 was completely abolished and the development of the tumor mass was not different from saline-treated mice (**Figure 7B**).

The toxicity of repeated treatments with NBs was assayed evaluating body weight and blood parameters such as lactate dehydrogenase (LDH), aspartate aminotransferase (AST), alanine aminotransferase (ALT), alkaline phosphatase, total creatinine kinase, creatinine, urea and also electrolytes. No side effects have been documented in the *in vivo* settings we have tested both NB2 and NB3 (data not shown).

## Discussion

Nanotechnology-based strategies for the specific delivery of microRNAs and antagomiRNAs have attracted much research (34, 35). Indeed, the nanocarriers can overcome the critical issues related to the administration of naked nucleic acids (36).

Among the number of nanodelivery systems, this project focused the attention on polymer-shelled nanobubbles as a tool to deliver nucleic acids. In the present study, we develop a new nanotherapeutic approach to deliver antagomiR17 using anti-CD20 targeted chitosan NBs for the treatment of aggressive B-NHL. In our approach, the therapeutic target is represented by miR-17, a key component of the miR-17~92 cluster, whose over-expression has been reported in several B-NHL, often in association with a worse clinical course (25, 28). Biologically, miR-17 has been reported to be involved in the regulation of cell cycle and of pro-apoptotic molecules, thus representing a good therapeutic target. For these purposes, an antagomiR17 molecule, able to bind and induce the degradation of miR-17, was developed (28). However, the therapeutic use of this antagomir17 molecule was demonstrated to be limited to intra-tumor injection, also in our aggressive B-NHL model. In fact, the local injection of antagomiR17 allowed only a partial reduction of miRNA level in tumor mass and we speculated that the remaining functional miR-17 molecules were able to sustain the development of the tumor mass. A systemic infusion is typically not approachable, mainly due to the low molecular weight, hydrophilicity, negative charge, and the enzymatic degradation of the molecule, as for all the small nucleic acids-based treatments. Indeed, the delivery of naked miRNA is associated with limitations such as short half-life in systemic circulation, inability to cross cell membranes and instability issues in biological fluids (37). Here, antagomiR17 was incorporated in purposely tailored anti-CD20 targeted decafluoropentane-cored chitosan NBs to increase its stability and to allow its selective delivery to cancer B-cells. This nanosystem is termed as “nanobubbles” for simplicity but “nanodroplets” would be more correct considering that decafluoropentane in the core is a perfluorocarbon liquid at room temperature.

Nanobubbles are spherical nanosized core/shell structures filled by a gas or vaporizable compounds (such as perfluorocarbons) and they have received increasing attention in drug, gas and gene delivery (38–40). Chitosan, a natural polycationic polysaccharide, was selected as component of NB shell for its biocompatibility, biodegradability, low toxicity, and no immunogenicity. Moreover, chitosan, with respect to other polymers, can form stable complexes with nucleic acids, exploiting electrostatic interactions between its positive charged amino groups and the negative charged phosphate groups of nucleic acids. All these features make chitosan an efficient compound for the development of nanocarriers aimed at the delivery of nucleic acids (41). The chitosan complexation capability has been exploited to design different nanocarriers such as nanoparticles, micelles, and nanocapsules (42, 43). Intriguingly, chitosan has been proposed for the manufacturing of polymer-shelled nanobubbles, showing a great binding capacity with siRNA and miRNA, due to the cationic properties of the shell (44, 45). Perfluoropentane has been selected as core component in all the preparations, being a perfluorocarbon liquid at room temperature (b.p. 29°C), to make easier the set up for nanobubble manufacturing than the one with a pressurized gas. Perfluorocarbons are components of ultrasound contrast agents and they have been studied as artificial oxygen carriers. Perfluoropentane is inert, biocompatible, eliminated by lung and accepted by the regulatory agencies, making the NB formulation a promising targeted tool for B-cell malignancies (46). Moreover, these nanosystems might allow the co-delivery of antagomiR17 with other drugs, considering the different domains of the NB structure (47, 48). This strategy can provide a synergistic mechanism of action thus increasing the cytotoxicity against tumor cells as previously suggested by our group (18).

Based on all the properties described above, chitosan-shelled NBs can be a versatile nanostructure with attractive advantages for the delivery of antagomir17. They can be produced with simple and scalable manufacturing process obtaining a good payload with high encapsulation efficiency. The nanosystem demonstrated physical stability up to 6 months and provided controlled release of the encapsulated antagomir17 after incubation in simulated cytosol. Moreover, the NB chitosan surface offers the possibility to be functionalized with specific target ligands for an active targeted delivery.

Different targeting and delivery strategies has been used to address BL cells, including folic acid (49), Pluronic F127 (23) and anti-CD20 antibodies. The latter approach was largely demonstrated to actively drive nanoparticles and drugs in the tumor microenvironment (18, 19, 21, 50), thus highlighting the relevance of this targeting mechanism.

The approach proposed in the present study is based on Rituximab, as anti-CD20 antibody driving chitosan-shelled NBs. We demonstrated *in vitro* a general binding and internalization of all NBs into B cells through phagocytosis, leading to the transport of antagomiR17 inside cells, with a consequent down-regulation of miR-17. Moreover, the data collected *in vitro* showed that both targeted and untargeted NB formulations have the capacity to bind tumor B-cells but the targeting agent (anti-CD20 antibody) is required to enhance the internalization of nanosystems. Chitosan-shelled NBs, injected in a human-mouse lymphoma model, also demonstrated the capacity to accumulate in tumor microenvironment in a very short period of time, maintaining their concentration for several days. Shape, size and surface charge of chitosan nanoparticles are key parameters that affect the accumulation in the tumor tissue (51, 52). For example, Mai et al. visualized the tumor-selectivity of cyanine 5.5-conjugated chitosan nanobubbles in a mouse tumor model by fluorescence imaging (53). Moreover, our previous studies have shown the NBs accumulation in tumor tissue with consequent reduction of tumor volume and weight in a human anaplastic thyroid cancer xenograft mouse model (54). On the contrary, other particles using the same targeting agent, previously studied in the same animal model (18), showed a different distribution pattern over time, a slower accumulation in the tumor microenvironment with a pick after 4 days. The difference was probably due to distinct physicochemical properties of the nanostructures, including the opposite charge between the 2 polymeric nanosystems. Interestingly, positively-charge chitosan-based NBs developed in this study allow the delivery of high concentration nucleic acid in less than 1 h. Both targeted and untargeted NBs accumulated in the tumor microenvironment as shown in optical imaging analysis, but we speculated that targeted NBs can be taken up by the tumor B-cells to a larger extent, as demonstrated *in vitro*.

Although the difference in the accumulation of untargeted and targeted NBs was not shown in biodistribution studies, the advantage of the targeting mechanism was particularly evident in the therapeutic efficacy studies. We have considered iv administration for distribution analysis, where a single injection is required. *In vivo* treatments often require several doses of therapeutics; for this reason, we preferred i.p. injections, creating less side effects in the mice to injections and volumes of the injections, as already demonstrated (16, 18, 22). In fact, no therapeutic effect was evidenced using untargeted NB loaded with antagomiR17, while the inhibition of tumor growth was clearly documented after repeated injections of targeted anti-CD20 NBs with antagomiR17. The effect is dependent by the presence of the anti-CD20 antibody but only for its driving capacity. Indeed, the targeted antagomir17-loaded NBs were ineffective in the treatment of a B-cell xenograft not expressing miR-17, showing also no cytotoxic effect of the nanosystem in the absence of the over-expression of miR-17. These data also demonstrated that the therapeutic effect was only due to the delivery of antagomiR-17 to cells and not to other component of the nanosystem; in particular, the low amount of Rituximab and its covalent binding on polymers did not allow the activation of the immune system and a direct cytotoxic effect. In general, anti-CD20 NBs loaded with antagomiR17 resulted safe and no side effects have been recorded during all the *in vivo* studies, even if it is important consider that anti-CD20 targeting agent is specific for human antigen and in a human context also normal B cells will be probably addressed by targeted nanoparticles. This characteristic also brought to the use of anti-CD20 antibodies in the treatment of some autoimmune diseases.

In conclusion, anti-CD20 targeted NBs loaded with antagomiR17 showed physicochemical stability and targeting properties suitable for antagomiR17 delivery both *in vitro* and *in vivo*. In fact, the nanoformulation was able to selectively target B-cell malignancies expressing CD20, thus leading to the release of antagomiR17 and inhibiting tumor growth in a B-cell lymphoma model.

## Data availability statement

The raw data supporting the conclusions of this article will be made available by the authors, without undue reservation.

## Ethics statement

The animal study was reviewed and approved by Italian Ministry of Health (Prot. N° 507/2016-PR).

## Author contributions

MD, RC, VG, RB and PM conceived the study. SC, MA, SB, TD’A, LD and DB performed the analyses. SC, MA, SB and TB generated plots and tables. MD, GT, RC, VG, RB and PM jointly supervised the project. SC and MA wrote the original draft. All authors participated in analyzing the data and writing the manuscript. All authors have read and agreed to the published version of the manuscript. All authors contributed to the article and approved the submitted version.
