# Supplementary material for: Targeted chitosan nanobubbles as a strategy to down-regulate microRNA-17 into B-cell lymphoma models
Source: Front Immunol. 2023 Jun 8;14:1200310. doi: 10.3389/fimmu.2023.1200310 (PMC10285521; doi:10.3389/fimmu.2023.1200310)
Supplement: Supplementary file 1 [file DataSheet_1.pdf]

Supplementary Material

Targeted chitosan nanobubbles as a strategy to down-regulate microRNA-17 into B-cell lymphoma models

Sara Capolla[1†], Monica Argenziano[2†], Sara Bozzer[1], Tiziana D'Agaro[3], Tamara Bittolo[3], Luigina De Leo[4], Tarcisio Not[4], Davide Busato[5], Michele dal Bo[5], Giuseppe Toffoli[5], Roberta Cavalli[2], Valter Gattei[3], Riccardo Bomben[3‡], Paolo Macor[1‡*]

[1] Department of Life Sciences, University of Trieste, 34127 Trieste, Italy

[2] Department of Scienza e Tecnologia del Farmaco, University of Turin, 10125 Turin, Italy

[3] Clinical and Experimental Onco-Hematology Unit, C.R.O.-IRCCS, 33081 Aviano, Italy

[4] Department of Pediatrics, Institute for Maternal and Child Health, IRCCS Burlo Garofolo, 34137 Trieste, Italy

[5] Experimental and Clinical Pharmacology Unit, C.R.O.-IRCCS, 33081 Aviano, Italy

[†] Equally contributed to the manuscript as "First author"

[‡] Equally contributed to the manuscript as "Senior author"

*** Correspondence:** Corresponding Author: pmacor@units.it

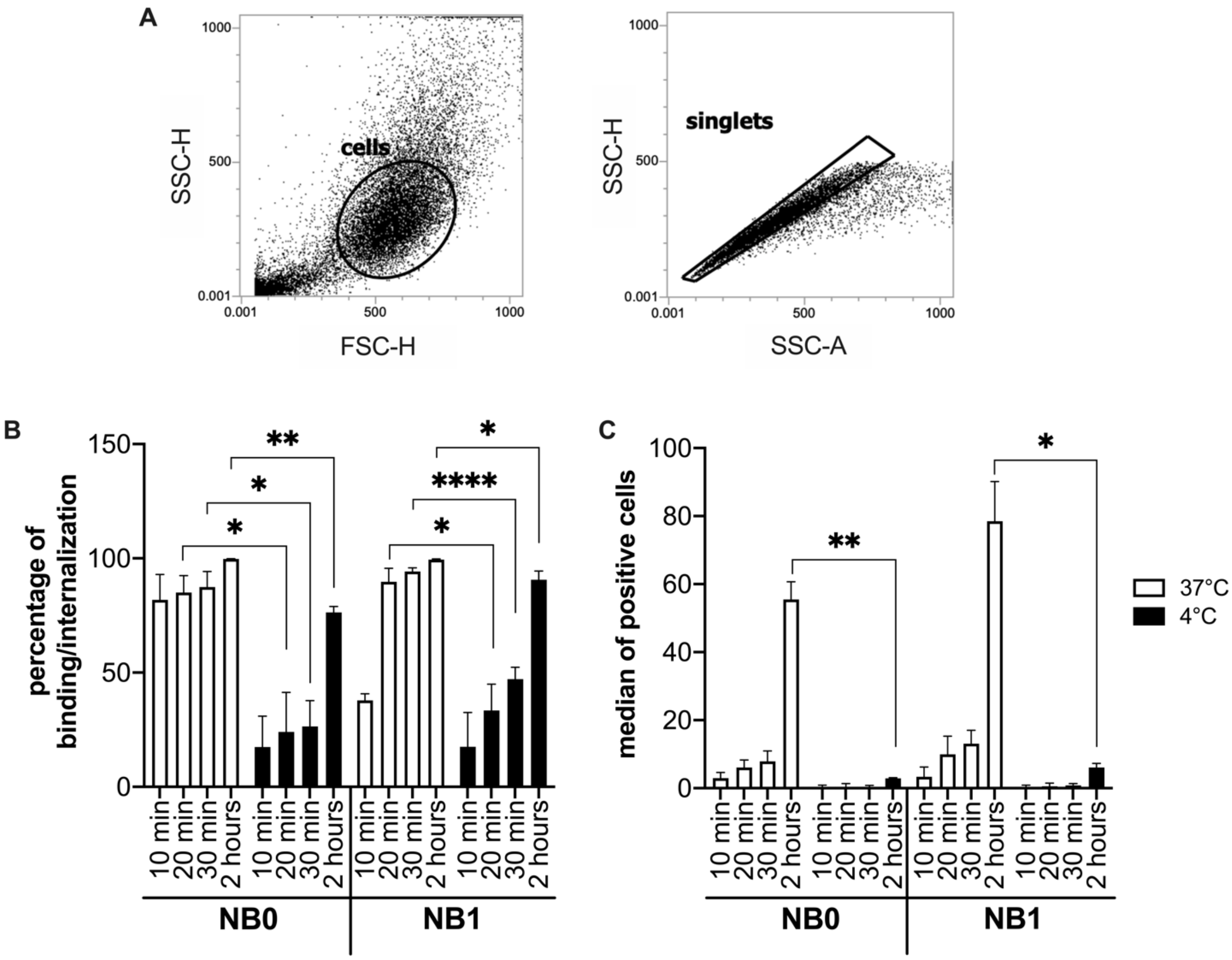

Supplementary Figure 1. NBs binding/internalization into macrophages is a membrane-mediated process. PMA-activated THP-1 cells were incubated with NBs overtime (10 to 120 minutes) at 37 or 4°C and analyzed by flow cytometry. **A.** The percentage of cells that bound/internalized NBs and **B.** the median fluorescence intensity of positive cells were analyzed. NB0: untargeted NBs; NB1: antiCD20-conjugated NBs. Data are showed as mean±SD. NB0 vs NB1: * P-value <0.05; ** P-value <0.005; **** P-value <0.0001.

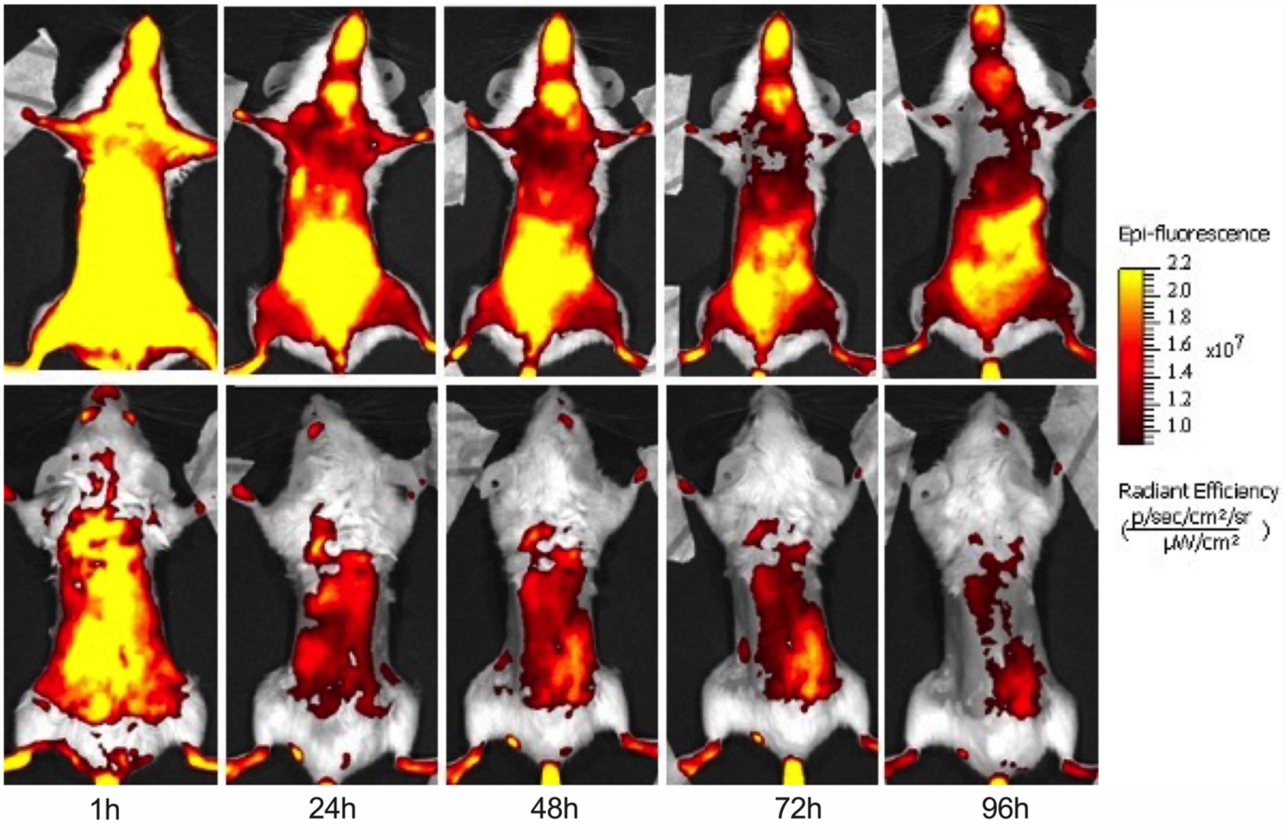

Supplementary Figure 2. Free Cy5.5 was mainly eliminated by the bladder. Mice received i.p. injection of BJAB cell. When a tumor mass was visible, 0.6nmol of free Cy5.5 were injected i.v. Mice were analyzed *in vivo* by the imager IVIS Lumina. Upper and lower pictures show a supine and prone mouse, respectively.
